# Faced with one’s fear: Attentional bias in anorexia nervosa and healthy individuals upon confrontation with an obese body stimulus in an eye‐tracking paradigm

**DOI:** 10.1002/brb3.1834

**Published:** 2020-09-12

**Authors:** AndreaSabrina Hartmann, Tiana Borgers, Jennifer Joanne Thomas, Claire‐Marie Giabbiconi, Silja Vocks

**Affiliations:** ^1^ Unit of Clinical Psychology and Psychotherapy Institute of Psychology Osnabrück University Osnabrück Germany; ^2^ Eating Disorders Clinical and Research Program Massachusetts General Hospital Harvard Medical School Boston MA USA

**Keywords:** anorexia nervosa, body dissatisfaction in women, body‐related attentional bias, eye‐tracking

## Abstract

**Objectives:**

Cognitive biases, particularly attentional biases, have been shown to be central to anorexia nervosa (AN). This study looked at attention deployment when consecutively viewing an obese and own body stimulus that both might represent feared stimuli in AN.

**Methods:**

Individuals with AN (*n* = 26) and mentally healthy controls (MHCs; *n* = 16) viewed a picture of themselves and a standardized computer‐generated obese body in random order for 4,000 ms each and then rated the attractiveness of the body parts of both stimuli. We compared dwell times on subjectively unattractive versus attractive body parts, and body parts that show weight status and gain most strongly (stomach, hips, thighs) versus least strongly.

**Results:**

For both stimuli, participants focused longer on the subjectively unattractive body parts (*p* < .01 and .001), with an even stronger attentional bias in individuals with AN regarding the obese stimulus (*p* < .05). Both groups also gazed longer at body parts indicative of weight status or gain (both stimuli *p* < .001), with no group differences.

**Conclusions:**

The attentional bias to one's own subjectively unattractive body parts might represent a mechanism maintaining body image disturbance in women in general. This attentional bias is even stronger when women with AN are confronted with an obese stimulus, highlighting a potential mental preoccupation with being fat or weight gain and a behavior distinct for the disorder.

## Graphical abstract

Both women with anorexia nervosa (AN) and healthy controls exhibited an attention bias toward subjectively unattractive body parts when viewing the own and an obese body stimulus. The bias was more pronounced in AN when confronted with the obese stimulus. Both groups also gazed longer at body parts indicative of weight status.

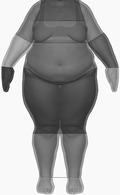

## 1 INTRODUCTION

Body image disturbance is a hallmark feature of anorexia nervosa (AN). It is characterized by a multifactorial pattern and dysfunctional attitudes and emotions toward one's body such as body dissatisfaction or fear of weight gain (Forrest, Jones, Ortiz, & Smith, 2018; Mitchison et al., 2017) as well as cognitive biases (Cordes, Bauer, Waldorf, & Vocks, 2015).

Body‐related cognitive biases are at the core of the model of negative body image by Williamson, White,York‐Crowe, and Stewart (2004). One bias concerns attention. Previous research indicates that biased attention toward body stimuli might even impact the development and maintenance of eating disorder (ED) symptoms (e.g., Dondzilo, Rieger, Palermo, & Bell, 2018) and is a mediator of the relationship between body mass index and body dissatisfaction (Porras‐Garcia et al., 2020). One method that has been repeatedly used to evaluate body‐related attentional bias is eye‐tracking, which assesses an individual's viewing patterns when confronted with body stimuli. Studies have demonstrated that body stimuli in general, and thin and fat body stimuli in particular draw increased attention in body‐dissatisfied compared to body‐satisfied individuals (for an overview: Rodgers & DuBois, 2016) as well as in individuals with AN, who, in addition, also show greater preoccupation with own body as opposed to others’ body‐related stimuli (for an overview: Ralph‐Nearman, Achee, Lapidus, Stewart, & Filik, 2019). Another share of research has investigated the viewing pattern across different parts of one body stimulus. In individuals with EDs, some (Bauer et al., 2017; Freeman et al., 1991; Jansen, Nederkoorn, & Mulkens, 2005; Roefs et al., 2008) although not all (von Wietersheim et al., 2012) studies have found that when viewing their own body, individuals with EDs fix their gaze longer on body parts they rate as unattractive than on those they rate as attractive. These findings are also corroborated by results from dimensional analyses indicating that increased body dissatisfaction is associated with a more pronounced deficit orientation when focusing on one's own body (Bauer et al., 2017; Roefs et al., 2008). Research in which participants view another (normal‐weight) control body is much more limited, but suggests a bias in the same direction, albeit less pronounced (Bauer et al., 2017). Furthermore, studies assessing differences in attentional bias for liked and disliked body parts between individuals with EDs (or high body dissatisfaction) and healthy individuals (with low body dissatisfaction) are inconsistent: While some studies indicated a bias for liked body parts in women without EDs as compared to women with EDs or high body dissatisfaction (Jansen et al., 2005), others found attentional bias for subjectively unattractive body parts in all women (Bauer et al., 2017; Freeman et al., 1991; Svaldi et al., 2016), in one study in healthy individuals only when positive mood was induced (Svaldi et al., 2016).

In addition to cognitive biases, emotions and attitudes—such as body dissatisfaction and fear of weight gain—have long been central to the phenomenology of AN and the main rationale assumed for food restriction (4th ed.; DSM‐IV; American Psychiatric Association [APA], 1994). However, the *Diagnostic and Statistical Manual of Mental Disorders* (5th ed.; DSM‐5; American Psychiatric Association [APA], 2013) removed fear of weight gain as the *sine qua non* of AN, which is in line with the assumption of a dimensional presentation of such symptoms in EDs (Olatunji et al., 2012). Given the unreliability of self‐report measures (Starzomska & Tadeusiewicz, 2016), especially with regard to the construct of fear of weight gain (Thomas, Hartmann & Killgore, 2013), eye‐tracking might prove useful by assessing attention deployment on body parts depending on whether they are an indication of weight status or gain (in the following shortened to weight status). Indeed, one study indicated that both healthy individuals and individuals with EDs attend more to areas that might indicate changes in weight status—either through showing body fat (e.g., stomach region) or making bones visible (e.g., collarbone)—when viewing pictures of another body, independent of its weight status (Horndasch et al., 2012). In a second study, this pattern only occurred in individuals with AN, but not healthy individuals (George, Cornelissen, Hancock, Kiviniemi, & Tovee, 2011).

Thus, in sum, it seems that individuals with EDs, particularly AN, show a bias to their subjectively unattractive body parts or body parts which indicate weight status most easily, both in pictures of themselves and of others. Findings hint at comparable but less pronounced biases in healthy control females. So far, however, the set of bodies presented to individuals with AN in order to analyze viewing patterns over different body parts has been narrow in terms of the weight spectrum, with overweight and obese bodies almost exclusively missing. Furthermore, assessments of viewing patterns which are potentially distorted toward areas indicating weight status have been limited to body stimuli of other persons.

Therefore, the present study sought to investigate whether compared to mentally healthy controls (MHCs), individuals with AN differ in their attention allocation to subjectively unattractive body parts and body parts which are most indicative of weight status, both in their own body and in an obese stimulus. In line with previous research, we expected women in both groups to show a greater attention allocation to their own body parts most indicative of weight status compared to less indicative body parts. Likewise, we expected all participants to show greater attention allocation to their subjectively unattractive body parts compared to their subjectively attractive body parts. This disparity was assumed to be even greater in the AN group. Given the unfortunate societal stigma toward obesity, which identifies overweight as a state to be avoided (e.g., Puhl & Heuer, 2009), we hypothesized that when confronted with an obese body stimulus, all women would focus longer on body parts most indicative of weight status and on subjectively unattractive body parts, but that this effect would be stronger in the AN group due to fear of weight gain. Furthermore, we expected that attentional bias to subjectively unattractive body parts and body parts most indicative of weight status would be positively correlated with restraint, as well as eating, weight, and shape concern in both groups and stimuli.

## 2 METHODS

### 2.1 Design

The study has a cross‐sectional quasi‐experimental design. Two groups, individuals with AN and MHCs, are compared on attention (dwell time in ms) allocated to the three subjectively most liked versus disliked body parts, and on attention allocated to body parts most versus least indicative of weight status, respectively, in a stimulus representing their own body as well as another computer‐generated obese body stimulus. Additionally, group‐specific associations of attention allocated to these body part clusters in both stimuli with restraint as well as eating, weight, and shape concern were examined.

### 2.2 Procedure

The study was approved by the local ethics committee. After the screening, participants provided written consent and completed two structured clinical interviews in the laboratory with the first author, who is a licensed clinical psychologist. Subsequently, a picture of the participants was taken, their height and weight were measured using a stable stadiometer seca 217 (seca) and bathroom scales Pino white 63747 (SOEHNLE), whereof body mass index (BMI) was calculated as kg/m^2^. Then, they took part in a Conjoint Analysis (Korn, Vocks, Thomas, Giabbiconi, & Hartmann, 2020) and an Implicit Association Test (IAT paradigm: T. Borgers, N. Krüger, S. Vocks, J. J. Thomas, F. Plessow, & A. S. Hartmann, unpublished data). After the eye‐tracking session, participants took part in an electroencephalography (EEG paradigm: A. T. Henn, T. Borgers, S. Vocks, C. ‐M. Giabbiconi, & A. S. Hartmann, unpublilshed data) and electromyography paradigm (EMG paradigm: A. S. Hartmann, unpublished data), and completed questionnaires in Unipark (QuestBack GmbH). Finally, the participants attended a debriefing session and received the reimbursement.

### 2.3 Participants

Participants with AN were recruited via treatment centers and online. MHCs were identified through online advertisements, local newspapers, and university mailing lists. Inclusion criteria included female gender, age ≥ 15 years, German‐language fluency, and no history of mania, psychosis, substance abuse, suicidal ideation, or eye conditions. Participants with AN had to have a body mass index (BMI) ≤18.5 kg/m^2^ and a DSM‐5 diagnosis of AN (APA, 2013). MHCs could not have experienced any mental health disorder and needed to have a BMI > 18.5 kg/m^2^. During the screening process, *n* = 51 and during clinical interviews *n* = 1 of 118 participants were found ineligible, leaving 66 participants of which data was collected. During the data‐cleaning process, data of *n* = 24 participants were lost. Of these, eye‐tracking data of nine participants were not recorded. A further 15 subjects were excluded due to lack of quality of eye‐tracking data. Of these, one participant had a tracking ratio of <70%, and another three participants presented with a deviation in *X* and/or *Y* higher than 1° that was not correctable. Finally, 11 of those 15 participants had to be excluded due to highly interrupted fixation movements visible in the scan paths, very late fixation beginnings and/or very low fixation durations in both variables (the summation of fixation durations for one's own and for the other body was lower than 1,000 ms) over the whole fixation period of 4,000 ms. This led to a remaining sample of *n* = 26 AN and *n* = 16 MHC. Participants received a reimbursement of 70 Euros for study participation.

### 2.4 Stimuli

**Figure 1:**
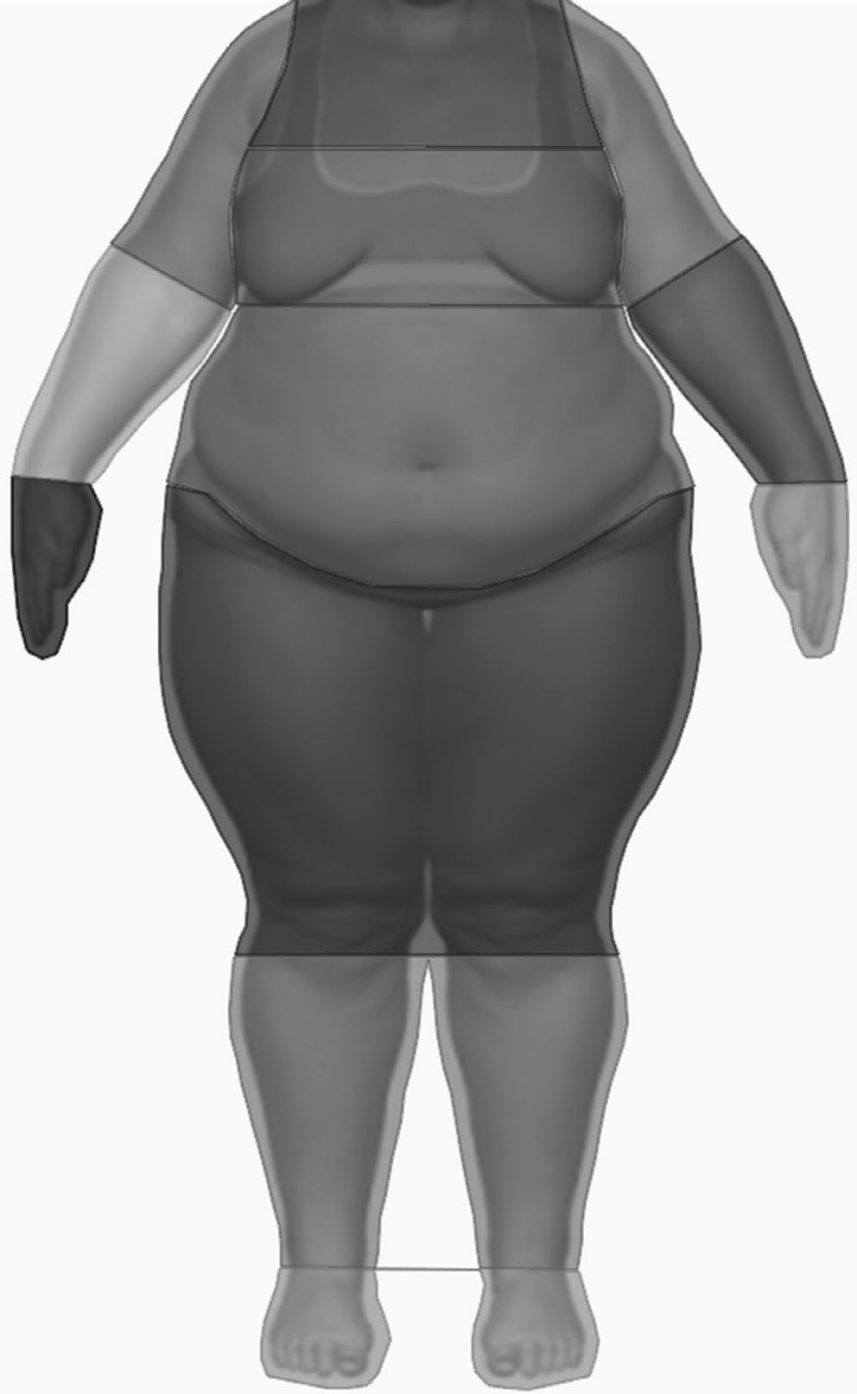
Computer‐generated obese body stimulus used in all participants with areas of interest (AOIs) drawn for analysis of viewing duration of different body parts. The AOIs corresponded to the body parts which participants were asked to rate in terms of attractiveness

One of the two stimuli comprised the picture taken of the participant wearing standardized gray underwear in their size, under standardized lighting conditions using a digital camera (Canon EOS 1200D with stigma 17–50 mm). The other stimulus was generated using the Rendering Software DAZ Studio 4.9 Pro. The 3D model Victoria 6.0 HD functioned as the basic figure and was rendered to represent an obese female body (see also Voges et al., 2017) wearing gray underwear comparable to the real one of the participants. Both stimuli were depicted in frontal view from the neck down, as the head would have drawn too much attention due to its relevance for social information (Hewig et al., 2008) and to enable comparison to previous studies which depicted stimuli in the same fashion (Bauer et al., 2017; Horndasch et al., 2012). Pictures were aligned in terms of body position, with the legs spread hip‐wide and arms spread to the side at a 45° angle. Figure 1 illustrates the obese stimulus, depicting the areas of interest (AOIs) that corresponded to the body parts which participants were asked to rate in terms of attractiveness (see instruments).

### 2.5 Eye‐tracking paradigm

A RED 500 eye‐tracker (SensoMotoric Instruments, SMI) was used, which is a remote, contact‐free (60–80 cm away from 22″ Dell monitor) eye‐tracking system providing an accuracy of 0.4°, a spatial resolution of 0.03° and a sampling rate of 500 Hz. In order to assess measurement accuracy, we conducted a five‐point calibration procedure (Experiment Center, SMI). In cases of low accuracy (maximum deviation of 0.5°; Holmqvist et al., 2011), calibration was repeated once. Pictures were presented five times for 4,000 ms each in a permuted order. During the inter‐trial interval (5,000 ms), a fixation cross was presented. Participants were instructed to freely view the pictures and, as a cover story, were told that the change in their pupil size would be recorded.

We prepared data in BeGaze (SMI) and mirrored procedures performed by Bauer et al. (2017). We computed dwell time (i.e., the sum of all valid fixations of 100 ms or more on one AOI) on subjectively attractive and unattractive body parts by summing dwell times for the three subjectively most attractive and unattractive body parts (i.e., AOIs) for both pictures (for a description of the rating procedure, see below). Dwell time on body parts most and least indicative of weight status were computed by summing dwell times on the stomach including hips and thighs (Horndasch et al., 2012) versus feet, calves, hands, underarms, and décolleté/shoulders. Fixations that did not fall on any of the above‐specified AOIs (i.e., body parts) or fell on the white space were excluded from the analyses. In order to mirror realistic presentation, we did not correct for AOI size.

### 2.6 Instruments

Anorexia nervosa and comorbid disorders were diagnosed using the Structured Clinical Interview for DSM‐IV (SCID; Wittchen, Zaudig, & Fydrich, 1997). Attractiveness of body parts was assessed by asking participants to rank‐order nine body parts (i.e., feet, calves, thighs, belly, breasts, décolleté and shoulders, upper arms, lower arms, hands; see also Bauer et al., 2017) according to attractiveness by placing them in order from most attractive to least attractive using a drag‐and‐drop procedure for both body stimuli separately. ED pathology was assessed using the expert interview Eating Disorder Examination (EDE, Hilbert & Tuschen‐Caffier, 2006).

### 2.7 Data analysis

To analyze the data, we used the Statistical Package for the Social Sciences SPSS 25 (IBM, Armonk, USA). Group differences in demographic and clinical variables were evaluated using analyses of variance (ANOVAs) or Kruskal–Wallis tests with post hoc *U* tests. For all subsequent tests, dwell time was the dependent variable. To test the hypotheses regarding participants’ body‐related attention allocation depending on attractiveness of the body parts, we conducted two 2 × 2 ANOVAs, one for each presented body stimulus (own vs. obese). The ANOVAs included the within‐subjects factor Attractiveness (subjectively attractive vs. unattractive body parts) and the between‐subjects factor Group (AN vs. MHC). To test the hypotheses regarding participants’ body‐related attention allocation depending on the body parts which are indicative of weight status, we again conducted two 2 × 2 ANOVAs with the within‐subjects factor Weight Status Relevance (indicative vs. not indicative of weight status) and the between‐subjects factor Group (AN vs. MHC). To evaluate the strength of the associations of the dysfunctional focus (i.e., dwell times on subjectively unattractive body parts and body parts most indicative of weight status in both stimuli) with restraint, as well as eating, weight, and shape concern, we computed Pearson's product‐moment correlation coefficients in both groups separately (*p* < .002). Effect sizes were interpreted as follows: *_p_η* ^2^ ≥ 0.01 small effect, *_p_η* ^2^ ≥ 0.06 medium effect and *_p_η* ^2^ ≥ 0.14 large effect; *r* ≥ .10 “small effect,” *r* ≥ .30 “medium effect,” *r* ≥ .50 “large effect” (Cohen, 1988).

## 3 RESULTS

### 3.1 Participant characteristics

The groups did not differ with respect to age. The AN group showed higher scores for ED psychopathology (see Table 1). Seven of the participants with AN (27%) had one comorbid diagnosis, and three (12%) had two comorbid diagnoses.

**Table 1:** Sociodemographic and clinical characteristics of AN and MHC groups

	AN (*n* = 26) *M* (*SD*)	MHC (*n* = 16) *M* (*SD*)	Group comparison
Age	23.28 (7.59) Min = 15 Max = 46	23.50 (2.66) Min = 19 Max = 28	*t* (40) = 0.45
Body mass index (kg/m^2^)	15.76 (2.70)	20.95 (2.44)	6.14***
Eating Disorder Examination—Questionnaire			
Restraint	3.08 (1.83)	0.10 (0.30)	−8.10***
Eating concern	2.50 (0.15)	0.05 (0.15)	−8.59***
Shape concern	3.39 (1.93)	0.51 (0.49)	−7.27***
Weight concern	2.70 (1.97)	0.60 (0.59)	−5.08***
Mean illness duration in years	8.83 (5.75)	–	

Abbreviations: AN, Anorexia Nervosa; MHC, Mentally Healthy Controls.

*** *p* < .001.

### 3.2 Attentional bias to subjectively unattractive body parts

The analyses of variance (ANOVAs) yielded a significant interaction effect of Attractiveness × Group for the obese body stimulus, but not for one's own body stimulus (see Table 2). Additionally, for both stimulus types, main effects of Attractiveness reached significance, with both groups focusing longer on subjectively unattractive versus attractive body parts. While there was no main effect of Group for one's own body stimulus, a significant main effect of Group for the obese body stimulus indicated significantly longer engagement in the AN group.

**Table 2:** Means and standard deviations and group differences in dwell times (ms) on subjectively attractive and unattractive body parts and body parts most and least indicative of weight status of AN and MHC groups

	AN (*n* = 26) *M* (*SD*)	MHC (*n* = 16) *M* (*SD*)	Group *F*(1, 40)/ *_p_η* ^2^	Attractiveness *F*(1, 40)/ *_p_η* ^2^	Group × Attractiveness *F*(1, 40)/ *_p_η* ^2^
Own body stimulus			0.12/<0.01	11.59**/ 0.23;	0.10/<0.01
Subjectively attractive body parts	554.40 (609.02)	564.09 (341.89)			
Subjectively unattractive body parts	1,202.96 (844.75)	1,103.68 (844.75)			
Other (obese) body stimulus			4.10*/ 0.09	64.78***/ 0.62	7.22*/ 0.15
Subjectively attractive body parts	212.20 (271.06)	0.87 (306.13)			
Subjectively unattractive body parts	1,900.27 (887.48)	1,235.14 (731.98)			

	AN (*n* = 26) *M* (*SD*)	MHC (*n* = 16) *M* (*SD*)	Group *F*(1, 40)/ *_p_η* ^2^	Weight Status Relevance *F*(1, 40)/ *_p_η* ^2^	Group × Weight Status Relevance *F*(1, 40)/ *_p_η* ^2^
Own body stimulus					
Body parts most indicative of weight	1,562.93 (901.65)	1,649.26 (888.58)			
Body parts least indicative of weight	39.13 (90.34)	107.67 (220.75)	0.31/ <0.01	103.98***/ 0.72	<0.01/ <0.01
Other (obese) body stimulus			0.60/ 0.02	64.61***/ 0.62	0.07/ <0.01
Body parts most indicative of weight	1,502.69 (952.92)	1,362.74 (634.98)			
Body parts least indicative of weight	230.63 (326.95)	170.13 (175.5)			

Abbreviations: AN, Anorexia Nervosa; MHC, Mentally Healthy Controls.

* *p* < .05

** *p* < .01

*** *p* < .001

### 3.3 Attentional bias to body parts indicative of weight status

We did not find a significant interaction effect of Weight Status Relevance × Group or a main effect of Group for either stimulus. However, both ANOVAs yielded a main effect of Weight Status Relevance (see Table 2). The findings indicated that both groups focused longer on body parts that are most indicative of weight status.

### 3.4 Correlations of eating disorder pathology with dwell times on body parts for both groups

No significant correlations emerged in the AN group, while dwell times on body parts indicative of weight status were related to eating concern and weight concern for the own body stimulus, and to eating concern and shape concern for the obese body stimulus in MHCs (see Table 3). Furthermore, dwell times on subjectively unattractive body parts in both stimuli were correlated with eating, weight, and shape concern in MHCs (see Table 3).

**Table 3:** Pearson product‐moment correlations (not corrected for multiple testing) of dwell times on subjectively unattractive body parts and those indicative of weight status with eating disorder pathology in the AN and the MHC groups, respectively

Dwell time (ms)	RE	EC	WC	SC
AN group				
Unattractive body parts in own body	0.29	0.15	0.10	0.13
Unattractive body parts in other (obese) body	<−0.01	0.02	−0.07	−0.07
Body parts indicative of weight status in own body	0.21	0.12	0.11	<−0.01
Body parts indicative of weight status in other (obese) body	−0.12	−0.12	−0.26	−0.36
MHC group				
Unattractive body parts in own body	0.12	0.72**	0.73**	0.75**
Unattractive body parts in other (obese) body	0.18	0.58*	0.56*	0.58*
Body parts indicative of weight status in own body	0.45	0.54*	0.71**	0.46
Body parts indicative of weight status in other (obese) body	0.22	0.60*	0.36	0.55*

Abbreviations: EC, Eating Concern; RE, Restraint; SC, Shape Concern of the Eating Disorder Examination; WC, Weight Concern.

* *p* < .05

** *p* < .01

## 4 DISCUSSION

The aim of the present study was to investigate whether individuals with AN, compared to MHCs, differ in their attention allocation to subjectively unattractive body parts and body parts which are indicative of weight status in their own body and in an obese stimulus. In line with our hypotheses regarding attention deployment depending on attractiveness of body parts, individuals with AN and MHCs deployed more attention to subjectively unattractive body parts for both stimuli. The viewing durations on these body parts were two to nine times longer than on attractive body parts. However, the expected interaction effect occurred only for the obese body stimulus; that is, the AN group focused even longer on the subjectively unattractive body parts relative to attractive ones. Furthermore, in line with our hypotheses regarding attention deployment depending on body parts that are indicative of weight status, both groups focused longer (7–40 times) on body parts most indicative of weight status in both stimuli.

Confirming previous research (Bauer et al., 2017; Freeman et al., 1991; Jansen et al., 2005), our study revealed self‐deprecating attentional biases in both AN and MHCs. One might argue that the current societal body ideal targets all women (Fardouly, Diedrichs, Vartanian, & Halliwell, 2015), which might lead to unrealistic expectations regarding one's own body and a consequent critical inspection of subjectively unattractive body parts. This assumption is supported by correlations of dwell time on subjectively unattractive body parts for the own body stimulus with measures of ED and body image pathology in MHCs.

For the obese body stimulus, we found an attentional bias to subjectively unattractive body parts, which was particularly pronounced in AN (nine times longer vs. three times longer [MHCs]). Given that attitudes toward obesity in the general population are often negative (Puhl & Heuer, 2009), it can be surmised that in both groups, this stimulus might represent a state to be avoided for oneself, and biases to subjectively unattractive parts of the obese body might reflect self‐motivational processes applied to avoid gaining weight (Pinhas et al., 2014). This is supported by the fact that the pronounced focus on subjectively unattractive body parts in the obese stimulus was positively associated with body image pathology in MHCs. The lack of corresponding associations in AN might be explained by dysfunctional cognitive processes having become independent in AN. However, it should be noted that for this stimulus, which is presumably a phobic stimulus for participants with AN, pronounced attention deployment to subjectively unattractive body parts might be a proxy for fear of weight gain (Thomas et al., 2013). This should be further explored by recruiting individuals with AN who explicitly report varying degrees of fear of weight gain (Olatunji et al., 2012).

In both stimuli and groups, we observed an attentional bias to body parts indicative of weight status but no difference between individuals with AN and MHCs, which corroborates earlier work (George et al., 2011; Horndasch et al., 2012). This outcome highlights the relevance of thinness for women irrespective of ED pathology (e.g., Fardouly et al., 2015).

The strongest limitation of our study pertains to the sample size, which was limited by the rigorous data‐cleaning process, as is common for eye‐tracking studies, but still slightly higher than the average 11%–25% data loss (Bauer et al., 2017; Tuschen‐Caffier et al., 2015). This might have led to a limitation of sample representativeness and bias. Furthermore, the low power might have led to some of the null findings in the ANOVAs. Therefore, we need to be cautious in interpreting null findings as a definite absence of group differences and recommend larger sample sizes in future studies. In line with previous studies (e.g., Bauer et al., 2017), we made use of a five‐point data calibration process. To decrease data loss in future studies, nine‐point or drift calibration should be used. Furthermore, despite the advantages of headless pictures, such as not leading to distraction (Hewig et al., 2008) and comparability with previous studies (Bauer et al., 2017; Horndasch et al., 2012), such pictures also come with limitations. First, they are not authentic (i.e., not what individuals see in the mirror). Second, there might be little identification with the stimulus when presented without a head. Given that identifying a body as one's own has been shown to alter the perception of its attractiveness (Voges et al., 2017), the unclear identification of individuals with headless bodies might have influenced participants’ attention deployment. And third, it might mask a differing exploration of underweight, normal‐weight, and overweight stimuli, as studies have shown that in normal‐weight stimuli (e.g., Leehr et al., 2018). Another limitation of our stimuli is the lack of a computer‐generated normal (or low) weight body as a control stimulus. We, therefore, need to be cautious with inferring fear of weight gain or other concepts from the viewing patterns across the obese body stimulus as these patterns could also arise as a consequence of the stimulus being unfamiliar in comparison with the own body stimulus. Additionally, we did not ask participants to rate attractiveness and pleasantness of the stimuli, which might have influenced their attention deployment pattern. Finally, as this is the first study to look at group differences in attention deployment processes regarding an obese stimulus in individuals with AN and MHCs, we decided against correction for multiple testing.

## 5 CONCLUSIONS

Taken together, this study demonstrated that women in general look longer at both subjectively unattractive body parts and body parts indicative of weight status. This bias to subjectively unattractive body parts is even more pronounced in women with AN when confronted with an obese body stimulus. Thus, it seems that obese stimuli—which might represent what individuals with AN fear the most—elicit a characteristic viewing pattern of body parts with different subjective attractiveness ratings, while women in general show a bias to unbalanced viewing patterns. Future studies might wish to focus on healthy women in order to understand the role of attention deployment to stimuli with various BMIs in the development of body dissatisfaction. Regarding clinical implications, prevention programs targeting body dissatisfaction in women might seek to target dysfunctional body‐related viewing patterns, particularly given their strong association with eating pathology in MHCs.

## CONFLICT OF INTEREST

The authors have no conflict of interests to declare.

## AUTHOR CONTRIBUTIONS

JJT, CMG, and ASH designed the study; CMG, SV, and ASH developed the paradigm. TB collected data supervised by CMG and ASH. TB and ASH analyzed and all authors interpreted data. TB and ASH drafted the first version of the article, and all authors critically revised the article and approved the final version.

### Peer Review

The peer review history for this article is available at https://publons.com/publon/10.1002/brb3.1834.

## Data Availability

The data that support the findings of this study are not publicly available, as the ethics committee stipulated that data must not be shared.
